# Ocular manifestations of Chikungunya fever in the chronic phase

**DOI:** 10.5935/0004-2749.20210081

**Published:** 2025-08-21

**Authors:** Louise Pellegrino Gomes Esporcatte, Arlindo José Freire Portes

**Affiliations:** 1 Department of Ophthalmology, Universidade Estácio de Sá, Rio de Janeiro, RJ, Brazil

**Keywords:** Chikungunya fever, Chikungunya virus, Dry eye syndrome, Eye infections, viral, Arbovirus infections, Eye manifestations, Febre Chikungunya, Vírus Chikungunya, Síndromes do olho seco, Infecções oculares virais, Infecção por Arbovírus, Manifestações oculares

## Abstract

**Purpose:**

To identify ocular manifestations in patients with Chikungunya fever in the chronic phase and describe their sociodemographic profile.

**Methods:**

Patients with serologic confirmation of Chikungunya infection were included in this transverse study. All subjects underwent a comprehensive ophthalmologic evaluation, including specific lacrimal function tests (tear break-up time test, Schirmer test, and lissamine green).

**Results:**

Overall, 64 eyes of 32 patients were evaluated. Most patients were women (71.9%), with the mean age of 50.0 ± 13.7 years. The mean interval between serologic confirmation and the examination was 12.7 ± 7.7 months. Twenty patients (62%) presented with dry eye. No statistically significant association was observed between dry eye and infection diagnosis time (*p*=0.5546), age (*p*=0.9120), sex (*p*=1.00), race (*p*=0.2269), arthralgia in acute infection (*p*=0.7930), retro-orbital pain (*p*=0.3066), and conjunctivitis (*p*=1.00).

**Conclusion:**

Dry eye was the most prevalent manifestation observed. No signs of intraocular inflammation and affected visual acuity were observed.

## Abstract (pt)

**Objetivo:**

Identificar manifestações oculares em pacientes na fase crônica da febre Chikungunya e descrever seu perfil sociodemográfico.

**Métodos:**

Estudo transversal com a inclusão de pacientes com confirmação sorológica de febre Chikungunya. Todos os pacientes foram submetidos a exame oftalmológico completo, incluindo testes específicos de função lacrimal (teste de ruptura do filme lacrimal, teste de Schirmer e teste da lissamina verde).

**Resultados:**

Foram avaliados 64 olhos de 32 pacientes. A maioria dos pacientes eram do sexo feminino (71,9%) e a idade média foi 50,0 ± 13,7 anos. O intervalo médio entre a confirmação sorológica e o exame oftalmológico foi de 12,7 ± 7,7 meses. Vinte pacientes (62%) apresentaram olho seco. Não houve significância estatística na associação entre olho seco e o tempo de diagnóstico da infecção (p=0,5546), idade (p=0,9120), sexo (p=1,00), raça (p=0,2269), artralgia durante a infecção aguda (p=0,7930), dor retro-orbitária (p=0,3066) e conjuntivite (p=1,00).

**Conclusão:**

A presença de olho seco foi a manifestação mais prevalente observada. Não foram observados sinais de inflamação intraocular ou baixa acuidade visual.

## INTRODUCTION

Chikungunya fever is a disease caused by the virus of the same name that affects the joints, and is potentially debilitating because of the intensity and chronicity of the pain. Transmission occurs through the female *Aedes aegypi* and *Aedes albopictus* mosquitoes infected by the Chikungunya virus (CHIKV), characterizing it as arboviruses^(1)^. The virus was first identified in 1952 in Makonde Plateau, southern Tanzania, during an epidemic, and its name in the native language means “the one who bends”-a reference to the antalgic posture of the infected person^(2)^.

Infected patients present in the acute phase (lasting 7 to 10 days) with high fever (>39°C), arthralgia, myalgia, and maculopapular rash. In addition, complaints of photophobia and retro-orbital pain are common. Approximately 15% of individuals are asymptomatic.^(3)^ The chronic phase is defined by symptoms, especially arthralgia and myalgia, that persist for more than 3 months^(1)^. CHIKV can cause ophthalmological symptoms that could be

Ocular manifestations of Chikungunya fever in the chronic phase present during the acute phase of the disease or occur several weeks after the disease onset^(3,4)^.

Ocular changes might occur because of the immune response associated with a hypersensitivity reaction. Notably, antibodies initiate the response against viral antigens responsible for joint involvement in systemic disease^(5)^. Nevertheless, the precise mechanisms of ocular involvement in Chikungunya fever have not yet been fully elucidated. Notably, the epithelial and endothelial cells of the cornea are the preferred targets of the virus^(6,7)^.

Ocular involvement of the CHIKV in the acute phase can manifest as conjunctivitis, retinitis^(4)^, choroiditis^(8)^, neuroretinitis, and even optic neuritis^(9)^. The most common finding described was bilateral anterior uveitis, often associated with increased intraocular pressure (IOP)^(10,11)^. The purpose of the current study was to identify ocular manifestations in patients with CHIKV and describe their sociodemographic profile in the chronic stage of the disease.

## METHODS

This transverse study was approved by the Institutional Ethics Committee of Estácio de Sá University, Rio de Janeiro, Brazil. It was performed per the ethical standards laid down in the Declaration of Helsinki and the International Conference on Harmonization Guidelines for Good Clinical Practice. Written informed consent was obtained from all participants before inclusion in the study. Patients were evaluated between May and October 2018.

Overall, 32 patients with laboratory confirmation of CHIKV infection were included in the study. CHIKV was serologically confirmed using the ELISA diagnostic method (Euroimmum Laboratory, SP, Brazil), which identified antibodies (IgM) against the virus and was collected by the Secretária de Estado de Saúde do Governo do Estado do Rio de Janeiro (LACENRJ).

All subjects underwent a comprehensive ophthalmologic examination, including best-corrected visual acuity (BCVA), manifest refraction, a pupillary reflex test, slit-lamp biomicroscopy, Goldmann applanation tonometry, gonioscopy, and a dilated fundoscopy examination with 78D fundus lens (Volk, Mentor, OH, USA). Subjects with corneal diseases, ocular trauma history, or those unable to cooperate with exams were excluded.

The infection phase was classified based on the date of the serologic evaluation and the date of ophthalmological evaluation. Subjects diagnosed for up to 1 year on the date of the ophthalmological examination were classified as having a recent infection and those diagnosed for more than 1 year were classified as chronic infection.

### Lacrimal function

The tear film break-up time (TBUT) was performed after instilling 1.0% fluorescein sodium with the blue cobalt filter, and a TBUT of <10 seconds was considered abnormal. The Schirmer test (ST) without anesthetic (ST type I) was performed according to the dry eye workshop guidelines^(12)^.

The ocular surface was assessed using lissamine green test, and the corneal and conjunctival staining was scored as described by the Copenhagen criteria^(13)^.

Notably, the diagnosis of dry eye was considered when TBUT or ST were positive.

### Statistical analysis

All statistical analyses were performed with Epi. info 7.2 (Centers for Disease Control, Atlanta, Georgia, USA). Frequencies were calculated, and Pearson’s chi-square test and ANOVA were employed for intergroup comparisons.

Demographic data were assessed based on the Instituto Brasileiro de Geografia e Estatística (IBGE) profile.

The intergroup comparison of the frequency of dry eye was performed based on age: <60 years and ≥60 years.

The alpha level (type I error) was set to 0.05.

## RESULTS

All patients presented with positive serology (IgM) for CHIKV between April 2016 and July 2018. Notably, 5 patients were infected in 2016, 17 in 2017, and 10 in 2018, and the mean age was 50.0 _±_ 13.7 years (range: 27-79 years). The mean interval between the infection confirmation and ophthalmologic evaluation was 12.7 ± 7.7 months (range: 0.9-27.8 months). Most patients were women (71.9%), with 59.4% belonging to the black race, and 31.2% having completed high school. Regarding the previous pathological history, 46.9% had systemic arterial hypertension and 15.6% diabetes mellitus type 2 (Table 1). No patient reported autoimmune disease, chronic kidney disease, liver disease, or glaucoma. One patient was pregnant at the time of the infection diagnosis.

**Table 1:** Demographic characteristics

Characteristics	n	Percentage (%)
Age groups		
<60 years	25	78.1
>60 years	7	21.8
Sex		
Female	23	71.9
Male	9	28.1
Race^*^		
Black	19	59.4
White	13	40.6
Systemic arterial hypertension	15	46.9
Diabetes Mellitus type 2	5	15.6

* Self-declared

Six patients presented with BCVA ranging from 20/25 to 20/60. The mean spherical equivalent was -0.27 ± 1.69 D (range: -7.00 to +2.50 diopters), and no patient had pupillary reflex defects.

Punctate keratitis was observed in three patients (9.4%) and cataract in seven patients (21.8%). No patient presented with previous or acute changes in the cornea, iris, or anterior chamber indicative of intraocular inflammation caused by the CHIKV. The mean IOP was 13.17 _±_ 3.10 mmHg (range: 8-30 mmHg). All subjects revealed an open angle on gonioscopic evaluation, without signs of previous or acute inflammatory activity.

Optic disc temporal atrophy was observed in four eyes (two patients) and inferior atrophy in one eye (one patient). A previous chorioretinitis scar was detected in two patients. Moreover, a small circular hypopigmentation area (<1/2 optic disc diameter) was observed in the nasal-inferior retinal quadrant in two patients. Of the 15 patients (46.9%) with systemic arterial hypertension, 12 (37.5%) presented with mild hypertensive retinopathy. Moreover, patients with diabetes exhibited no signs compatible with diabetic retinopathy.

The most frequently reported symptoms at the beginning of infection were joint pain (96.9%), fever (93.7%), body pain (81.2%), headache (71.9%), and retro-orbital pain (59.4%). Based on the lacrimal function evaluation, 20 patients (62%) presented with dry eye. Excessive evaporation type (evaluated by TBUT) was observed in 45% of patients, whereas 55% presented with dry eye based on both excessive evaporation and aqueous deficiency (assessed using ST). The ocular surface staining was altered in six eyes of four patients.

No statistically significant intergroup differences were observed based on the infection time (recent or chronic infection, *p*=0.5546).

Furthermore, no statistical difference was observed regarding the frequency of dry eye diagnosis per the age-based classification (*p*=0.9120). The mean age of patients with and without dry eye was 53.3 ± 12.8 years and 44.5 ± 13.9 years, respectively (ANOVA, *p*=0.0779).

No statistically significant correlation was observed between dry eye and sex (*p*=1.00), race (*p*=0.2269), arthralgia in acute infection (*p*=0.7930), retro-orbital pain (*p*=0.3066), or conjunctivitis (*p*=1.00) (Table 2).

**Table 2:** Correlation between the presence of dry eye and risk factors

Characteristics	n (%)	OR (95% Cl)	*P value*
Infection time			
Recent	5 (50.00)	1	0.5546
Chronic	15 (68.18)	2.1429 (0.439-9.8982)	
Age			
>60 years	5(71.43)	1	0.9120
<60 years	15 (60.00)	0.6 (0.0968-3.7204)	
Gender			
Male	6 (66.67)	1	1.0
Female	14(60.87)	0.7778 (0.154-3.9273)	
Race^*^			
Black	14(73.68)	1	0.2269
White	6 (46.15)	0.3061 (0.0687-1.3636)	
Arthralgia			
Yes	20 (64.52)	1	0.7930
No	0 (0.00)	indefinite	
Retro-orbital pain			
Yes	10(52.63)	1	0.3066
No	10(76.92)	0.333 (0.0691-1.6077)	
Conjunctivitis			
Yes	2 (50.00)	1	1.0
No	18 (64.29)	0.556 (0.0676-4.5683)	

OR= *odds ratio*; CI= confidence interval;

* = Self-declared.

## DISCUSSION

Few previous studies have described ophthalmological changes because of Chikungunya fever. However, the existing literature consists mainly of case reports or retrospective studies of patients seeking emergency care for an acute low vision or ophthalmologic complaint. A retrospective study performed in India revealed that the onset of ocular manifestations ranged from 4 to 12 weeks (mean of 6 weeks) after the acute disease onset^(11)^.

In the present study, 4 patients (12.5%) reported conjunctivitis, and 10 reported retro-orbital pain (59.3%) in the acute period of infection. In a retrospective study, conjunctivitis was reported in 27 patients (19.4%); however, this diagnosis was performed by the emergency physician, who was not an ophthalmologist^(14)^. A review of ocular manifestations caused by the CHIKV reported that photophobia, conjunctival hyperemia, and retro-orbital pain were frequent findings during the acute phase of the disease and might be present without other ophthalmological alterations^(15)^.

In our study, during the acute phase of infection, 31 patients reported joint pain (96.9%), 30 fever (93.7%), 26 body pain (81.2%), 23 headaches (71.9%), 11 skin rash (34.4%), and 6 nausea (18.7%). Notably, these syste mic symptoms are compatible with those described in the literature. Notably, Mahendradas et al. reported that nine patients had fever, headache, nausea, and vomiting associated with arthralgia and skin rash^(11)^.

Nevertheless, we observed variation in BCVA between 20/20 and 20/60. A retrospective study of a series of 37 cases conducted in 2007 in India revealed that the visual acuity varied between 20/20 and light perception. However, the patients were evaluated for only 3 months after the diagnosis of CHIKV infection^(8)^. Another study observed that the BCVA ranged from 20/40 in 11 eyes (61.1%) to worse than 20/200 in 2 eyes (11.1%)^(9)^.

In our study, dry eye was diagnosed in 20 patients (62%) through lacrimal function tests. Among patients with a dry eye diagnosis, the type characterized by excessive evaporation was observed in 45%, whereas 55% presented dry eye featuring both excessive evaporation and aqueous deficiency. Nonetheless, no statistically significant association was noted between dry eye and age. In the Dry Eye Workshop (DEWS II), the prevalence of dry eye was reported to range from 5% to 50%, increasing significantly with age, exhibiting a linear association^(12)^.

Although dry eye syndrome is more prevalent in older patients, our study did not observe statistically significant differences related to the age of patients with or without dry eye. Therefore, we can conclude that age did not influence the presence of dry eye in this study. A cross-sectional study by Castro et al. evaluated the prevalence of dry eye in the five regions of Brazil. They observed that in the southeast, the prevalence of dry eye was 11.13%^(16)^. However, no statistically significant diffe rence was observed dry eye and other risk factors for its occurrence described in the literature, such as sex, race, or factors inherent in viral infection, such as the time of onset and symptomatology in the acute phase of the disease. Specific examinations to evaluate dry eye, such as TBUT, ST, and the lissamine green test, were not described in the literature. This study observed punctate keratitis in three patients during the chronic phase of the disease. Furthermore, Lalitha et al. observed three patients with keratitis characterized by the dendritic pattern, similar to that caused by the herpes virus^(8)^.

Nevertheless, because Chikungunya fever is a disease that characteristically affects the joints, it is essential to evaluate the association between rheumatologic factors and the tear film. A prospective case-control study conducted in Japan in 2003 involving 72 patients concluded that dry eye is common in patients with rheumatoid arthritis (92%), despite no association with Sjögren’s syndrome (90%)^(17)^.

Notably, viruses can trigger autoimmune reactions through several mechanisms, affecting various tissues. Once the innate immune response is initiated, tissue damage and dysfunction can occur because of apoptosis and inflammation. Therefore, the presence of a Sjögren syndrome-like illness, reported as signs or symptoms of dry eye, was observed in infections caused by the human T-cell lymphotropic virus, human immunodeficiency virus, Epstein-Barr virus, hepatitis C virus, and herpes virus^(18)^.

In this study, no signs related to previous intraocular inflammation were observed in the corneal endothelium, iris, anterior chamber, or angle. However, we cannot rule out the occurrence of acute uveitis during the early stage of the disease, which did not leave sequelae, such as anterior or posterior synechiae and keratic precipitates, in the corneal endothelium. In a retrospective study, five patients presented with iridocyclitis during the acute phase of the infection without alterations observed on gonioscopy or fundoscopy ^(11)^. Lalitha et al. described 11 patients with anterior uveitis (10 non-granulomatous and 1 granulomatous), but these cases were diagnosed during the period up to 3 months after the acute phase of infection^(8)^.

Notably, fundoscopy examination revealed chorioretinitis scarring in two patients, which could be a sequela of CHIKV infection or other causes of posterior uveitis, such as toxoplasmosis, which is the most prevalent uveitis in Brazil^(19)^. Moreover, the optic disc atrophy seen in three patients could be a sequela of optic neuritis. After up to 3 months of infection, Lalitha et al. observed four patients with optic neuritis, one with bilateral neuroretinitis, two with retinitis and vitreitis, and two with multifocal choroiditis and macular edema. They concluded that patients had an excellent visual prognosis because most of them did not have visual sequelae.^(8)^ However, it was not possible to rule out the occurrence of these changes in our study sample because we did not include the acute charts.

Nonetheless, our study had several limitations. First, we evaluated a small sample, and the evaluation was performed after the resolution of the acute phase. Therefore, patients with positive serology for Chikungunya fever could have presented with ocular manifestations during the acute phase of the disease, which could not be detected using this protocol. Moreover, several patients with positive serology could not be contacted (change of telephone contact) or did not attend the scheduled appointments. Therefore, it is impossible to rule out a selection bias in our sample because there is a possibility that patients participating in the study might have been seen because of their eye symptoms. Our study did not employ a dry eye questionnaire regarding the symptoms of the disease because the dry eye diagnosis was a result of a comprehensive eye examination in the study and not the focus of it.

In conclusion, dry eye was the most frequent manifestation observed during ophthalmological evaluation of patients with serologically-confirmed CHIKV. Nevertheless, we did not observe statistically significant associations between dry eye and other characteristics previously described in the literature. Nonetheless, further studies are required to assess the relationship between chronic articular disease caused by Chikungunya fever and dry eye disease.
